# Brushing Away the Blues: Self-Reported Oral Hygiene Practices Are Associated With Mild Depressive Symptoms in Airline Pilots

**DOI:** 10.7759/cureus.60695

**Published:** 2024-05-20

**Authors:** Piercarlo Minoretti, Miryam Liaño Riera, Manuel Gómez Serrano, Andrés Santiago Sáez, Ángel García Martín

**Affiliations:** 1 Occupational Health, Studio Minoretti, Oggiono, ITA; 2 Legal Medicine, Psychiatry, and Pathology, Complutense University of Madrid, Madrid, ESP; 3 Legal Medicine, Hospital Clinico San Carlos, Madrid, ESP

**Keywords:** flight safety, occupational health, mouthwash, toothbrushing, oral hygiene, depression, airline pilots

## Abstract

Introduction

Airline pilots are susceptible to mental health issues, with depression prevalence ranging from 1.9% to 12.6%. Recent research in the general population indicates a potential link between depression and oral health. In this cross-sectional study, we sought to investigate the association between self-reported oral hygiene practices and depressive symptoms among airline pilots.

Methods

One hundred actively working male airline pilots of Caucasian descent voluntarily enrolled in the study during routine occupational health visits. Depressive symptoms were assessed using the Beck Depression Inventory II (BDI-II). Self-reported oral hygiene practices, including toothbrushing frequency and mouthwash usage, were examined. Univariable and multivariable logistic regression analyses were performed to investigate associations between depressive symptoms and oral hygiene practices.

Results

Twelve pilots (12%) demonstrated mild depressive symptomatology (BDI-II scores 14-19). Pilots with mild depression reported significantly lower rates of brushing teeth twice or more per day (33.3% vs. 80.7%) and higher rates of rarely brushing (16.7% vs. 1.1%) compared to those with minimal depressive symptoms (p < 0.001). Nonuse of mouthwash was more prevalent among pilots with mild depression (66.6% vs. 23.9%, p = 0.008). Multivariable logistic regression analysis revealed that pilots who rarely brushed their teeth (adjusted odds ratio (OR) = 14.6; 95% confidence interval (CI) = 1.3−197.9; p < 0.05) or did not use mouthwash (adjusted OR = 5.7; 95% CI = 1.4−25.2; p < 0.05) had significantly higher odds of mild depressive symptoms.

Conclusions

Self-reported oral hygiene habits may serve as a proxy indicator for mild depressive symptoms among airline pilots. Incorporating oral health assessments into routine aeromedical examinations could provide a practical method of identifying pilots at risk for depression, supporting timely interventions and enhancing flight safety.

## Introduction

Airline pilots are entrusted with the critical responsibility of ensuring the safe travel of millions of passengers worldwide [1]. Despite their pivotal role, pilots are not immune to mental health challenges, with reports indicating a prevalence of depression between 1.9% and 12.6% [2]. The implications of depressive symptoms, which can impair cognitive functions and decision-making skills [3], are particularly concerning in aviation due to the potential impact on flight safety [4,5]. Consequently, identifying and addressing depressive symptomatology in airline pilots is of utmost importance.

Several recent studies have suggested an association between depression and oral health in the general population [6-9]. Intriguingly, a study by Anttila et al. [10] has reported that individuals with a higher number of depressive symptoms tend to brush their teeth less frequently and visit the dentist less often than those with fewer or no symptoms. Conversely, maintaining good oral health has been identified as a protective factor against depressed mood [11]. Despite these findings, there is a surprising scarcity of research exploring the specific relationship between oral hygiene and depression among airline pilots. This gap is noteworthy considering the unique challenges faced by this professional category, such as erratic work schedules and lifestyle disruptions, which could compromise their oral care routines [12]. Nonetheless, the potential for oral hygiene habits to provide an unexpected window into the presence of depressive symptoms is particularly relevant within an aeromedical context. Accordingly, the aviation industry has faced challenges in openly addressing mental health concerns among its workforce, which can deter pilots from seeking help or reporting mental health issues during medical evaluations [13]. In addition, pilots might fear the professional consequences of a depression diagnosis, worrying that it could result in the suspension of their medical certification, a process that can be both time-consuming and expensive to reverse [14].

Given these challenges, we designed the current cross-sectional study to determine whether self-reported oral hygiene behaviors, such as the frequency of toothbrushing and the use of mouthwash, are independently associated with the presence of depressive symptoms in airline pilots. The findings could have significant implications for the early identification and intervention of depression in this occupational group, potentially enhancing both pilots’ well-being and the safety of air travel.

## Materials and methods

Participants

The current study was conducted at outpatient clinics (Studio Minoretti, Oggiono, Italy) during routine occupational health visits. The participants were volunteers who agreed to participate in the research, and no random sampling was performed. An occupational health physician was responsible for extending the invitation to participate in the investigation. The study sample and procedures have been previously described in detail [3]. In brief, 100 actively working male airline pilots of Caucasian descent voluntarily enrolled in the study. Due to the underrepresentation of female pilots, the study focused exclusively on male subjects. The inclusion criteria required participants to be free from any known history of neurological, psychiatric, inflammatory, autoimmune, or infectious diseases. Additionally, individuals who had been diagnosed with cancer or had taken any medications within the past 90 days were excluded from the study. None of the participants were consuming dietary supplements, and all were deemed to be in satisfactory physical health. Prior to the commencement of the study, each pilot provided their written informed consent. The research was conducted in accordance with the ethical guidelines outlined in the Declaration of Helsinki and received approval from the local ethics committee (Studio Minoretti reference number: 2021/04E).

Assessment of depressive symptoms

We investigated the presence of depressive symptoms among airline pilots using the Beck Depression Inventory II (BDI-II), a widely used and validated self-reporting instrument [15]. The BDI-II consists of 21 items that assess both the presence and severity of depressive symptoms. The scores obtained from this tool are categorized into four ranges, each representing a different level of depressive severity. Scores ranging from 0 to 13 indicate minimal depressive symptoms, while scores between 14 and 19 suggest mild depression. Scores from 20 to 28 are indicative of moderate depression, and scores falling between 29 and 63 signify severe depression [3, 15].

Oral hygiene practices

We examined two self-reported oral hygiene practices: (1) toothbrushing frequency [16] and (2) mouthwash usage [17]. To assess toothbrushing frequency, the airline pilots were asked, “During a typical day in the last 12 months, how frequently did you brush your teeth?” The responses were categorized into three groups: brushing twice or more per day, once per day, or rarely. Regarding mouthwash usage, the participants were classified as habitual users, sporadic users, or nonusers based on their reported habits.

Data analysis

As none of the airline pilots in the current study achieved a score of 20 or higher on the BDI-II, the sample was divided into two categories based on established threshold scores. The first group, scoring between 0 and 13, was indicative of minimal depressive symptoms, whereas the second group, with scores ranging from 14 to 19, represented mild depression [3]. Categorical data were reported as counts and percent frequencies, and the Fisher's exact test was employed to compare these variables between the two groups. Continuous variables were expressed as means ± standard deviations and compared using unpaired Student’s *t*-tests. The associations between the presence of mild depressive symptoms and oral hygiene practices were investigated using both univariable and multivariable logistic regression analyses. The multivariable model was adjusted for potential confounding factors, including age, body mass index (BMI), and education levels. The results are presented as odds ratios (ORs) along with their corresponding 95% confidence intervals (CIs). Statistical analyses were performed using the IBM SPSS Statistics for Windows, Version 20 (Released 2011; IBM Corp., Armonk, New York, United States). All tests were two-tailed, and a p value < 0.05 was considered statistically significant.

## Results

Participant characteristics

The majority of pilots included in the study (n = 88; 88%) exhibited minimal depressive symptoms, with BDI-II scores ranging from 0 to 13. The remaining 12 (12%) of the sample demonstrated mild depressive symptomatology, as indicated by BDI-II scores between 14 and 19. The general characteristics of airline pilots, categorized by the severity of depressive symptoms, are presented in Table 1. There were no significant differences in age, BMI, or educational levels between the two groups.

**Table 1:** General characteristics of airline pilots categorized by the severity of depressive symptoms BMI: Body mass index; BDI: Beck Depression Inventory; ns: not significant Categorical variables are presented as frequencies (percentages) and compared using Fisher's exact test. Continuous variables are expressed as means ± standard deviations and compared using independent sample *t*-tests.

	Pilots with mild depression	Pilots with minimal depressive symptoms	P
	(n = 12)	(n = 88)	
Age, years	40 ± 5	39 ± 4	ns
Male sex, n (%)	12 (100)	88 (100)	ns
BMI, kg/m^2^	24 ± 2	23 ± 3	ns
Education, years	17 ± 2	17 ± 3	ns
BDI-II score	16 ± 2	5 ± 3	<0.001

Oral hygiene behaviors

Table 2 presents a comparative analysis of self-reported oral hygiene practices among airline pilots, categorized based on the severity of depressive symptoms. Regarding brushing frequency, only 33.3% of pilots with mild depression reported brushing their teeth twice or more per day compared to 80.7% of those with minimal depressive symptoms. Furthermore, 50% of pilots with mild depression brushed their teeth once per day, a rate significantly higher than the 18.2% observed in the minimal symptom group. Moreover, 16.7% of pilots with mild depression reported brushing rarely compared to only 1.1% of those with minimal depressive symptoms. These differences in brushing frequency were found to be statistically significant (p < 0.001). In terms of mouthwash use, 16.7% of pilots with mild depression were habitual users, a rate lower than the 28.4% observed in pilots with minimal depressive symptoms. Sporadic use of mouthwash was reported by 16.7% of pilots with mild depression and 47.7% of those with minimal symptoms. Notably, a significant proportion of pilots with mild depression (66.6%) did not report using mouthwash, compared to 23.9% in the minimal symptoms group (p = 0.011).

 

**Table 2:** Self-reported oral hygiene practices of airline pilots categorized according to the intensity of depressive symptoms Variables are presented as frequencies (percentages) and compared using Fisher's exact test.

	Pilots with mild depression	Pilots with minimal depressive symptoms	p value
	(n = 12)	(n = 88)	
Frequency of toothbrushing, n (%)			
Twice or more per day	4 (33.3)	71 (80.7)	<0.001
Once per day	6 (50.0)	16 (18.2)	
Rarely	2 (16.7)	1 (1.1)	
Use of mouthwash, n (%)			
Habitual users	2 (16.7)	25 (28.4)	0.011
Sporadic users	2 (16.7)	42 (47.7)	
Nonusers	8 (66.6)	21 (23.9)	

Univariable and multivariable logistic regression analyses

**Figure 1:**
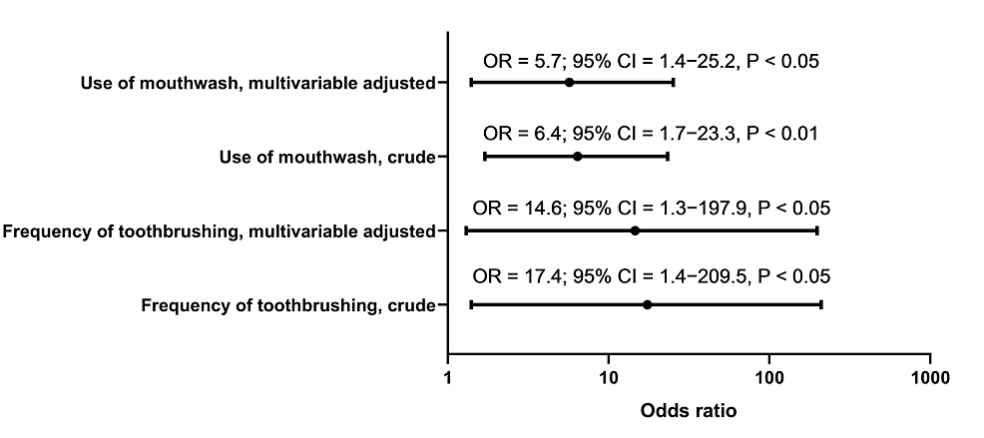
Odds ratio (Forest) plot showing the associations of self-reported oral hygiene practices with mild depression in airline pilots The odds ratios reported on the x-axis are displayed on a logarithmic scale.

In univariable logistic regression analysis, pilots who reported rarely brushing their teeth were more likely to experience mild depression compared to those who brushed their teeth at least once per day (crude OR = 17.4; 95% CI = 1.4−209.5; p < 0.05). Similar findings were observed for pilots who did not report using mouthwash compared with those who used mouthwash sporadically or habitually (crude OR = 6.4; 95% CI = 1.7−23.3; p < 0.01). The results did not change appreciably after adjusting for age, BMI, and education levels in a multivariable logistic regression analysis (frequency of toothbrushing: adjusted OR = 14.6; 95% CI = 1.3−197.9; p < 0.05; use of mouthwash: adjusted OR = 5.7; 95% CI = 1.4−25.2; p < 0.05) (Figure 1).

## Discussion

In this cross-sectional study, we observed that airline pilots who reported infrequent toothbrushing or lack of mouthwash use had significantly higher odds of exhibiting mild depressive symptoms compared to those with more optimal oral hygiene habits, even after adjusting for potential confounding factors. These findings may have relevant implications in the field of occupational medicine, as they highlight the potential utility of oral hygiene habits as a proxy indicator for the presence of mild depression among airline pilots. This is of particular relevance given the previously reported association between mild depression and impaired executive functioning in this occupational group [3].

Our results, obtained in a highly specific occupational setting, align with previous research in the general population indicating that poor oral health behaviors are frequently observed in individuals with depressive symptoms. A systematic review by Stepović et al. [6] identified several barriers affecting the oral health of people diagnosed with depression, including lack of motivation, side effects, and limited access to dental care. Using the data from the National Health and Nutrition Examination Survey 2015-2016, Almohaimeed et al. [7] found that individuals with depression had a higher prevalence of untreated dental caries and periodontal disease compared to those without depression. Tiwari et al. [8] explored the association between mental health, oral health status, and care utilization using a large, nationally representative sample. The results showed that individuals with poor mental health were more likely to have untreated dental caries, missing teeth, and lower utilization of dental services. A study by Cui et al. [9] investigated the relationships among oral hygiene behavior, toothache, and depressive symptoms in Chinese older adults. The authors found that poor oral hygiene behavior and toothache were associated with higher levels of depressive symptoms, suggesting a bidirectional relationship between oral health and mental health [9]. Anttila et al. [10] showed that individuals with depressive symptoms were more likely to have poor oral hygiene habits and perceive a need for dental treatment. In addition, a longitudinal study by Zwick et al. [11] found that poor oral health-related quality of life was associated with an increased risk of developing depressive symptoms over time. Collectively, these data suggest that the relationship between depression and oral health is complex and bidirectional.

In the context of airline pilots, who face unique occupational stresses and irregular schedules [18], maintaining regular oral hygiene routines might be particularly challenging, potentially exacerbating this issue. However, considering the cross-sectional design of our study, which is unable to measure causes and effects, we acknowledge that the observed relationship is not necessarily causal. One of the main implications of our findings is that including questions about oral hygiene habits in routine aeromedical assessments could provide a valuable screening tool for identifying pilots at risk of depressive symptoms, thereby enhancing early intervention strategies. The Germanwings tragedy highlighted the need for the aviation industry to better address mental health concerns among its workforce [2]. However, pilots remain apprehensive about a diagnosis of depression due to fears that it could jeopardize their professional future and ability to continue flying [13]. Consequently, they may be hesitant to openly disclose mental health challenges to their employers or regulatory authorities. Under these circumstances, as oral hygiene practices are less stigmatized and can be more readily disclosed, they could serve as a noninvasive proxy indicator of a pilot's depressive symptoms. By paying greater attention to oral health indicators, aviation medical examiners may be able to detect signs of depression that pilots are reluctant to report, providing more opportunities for timely intervention and treatment to mitigate risks to pilot well-being and flight safety.

Despite these promising findings, several methodological limitations in this study should be acknowledged. Although self-reported toothbrushing frequency is considered a valid proxy measure of oral hygiene practices [16], other indicators and potential confounders (e.g., dental visits, professional teeth scaling, and underreported alcohol use) were not accounted for. Due to the retrospective assessments of exposure, information bias cannot be ruled out. The study sample was limited to male Caucasian airline pilots, which may limit the generalizability of the findings to other ethnic groups or professionals working in different geographic locations. Future research should aim to include a more diverse demographic to enhance the external validity of the findings. Moreover, studies in airline pilots should adopt a longitudinal design to explore whether changes in oral hygiene habits can predict shifts in depressive symptoms over time or vice versa. In addition, qualitative studies could further enrich our understanding by capturing the personal experiences of this professional group regarding the interplay between their oral and mental health. Finally, another promising avenue for future research is to examine the potential association between barodontalgia, a condition characterized by oral pain triggered by changes in barometric pressure [19], and depressive symptoms among airline pilots. Investigating this link could shed light on the specific occupational factors that may contribute to the mental health burden in this population and inform targeted interventions to support their well-being.

## Conclusions

This study highlights the potential of self-reported oral hygiene habits to serve as a proxy indicator for mild depressive symptoms among airline pilots. Incorporating oral health assessments into routine aeromedical evaluations may provide a practical and less stigmatizing method of identifying pilots who may be at risk for depression, thereby supporting timely and appropriate interventions. This approach not only has the potential to improve individual health outcomes but also enhances overall flight safety by ensuring the well-being of pilots.
